# Availability of Illegal Drugs During the COVID-19 Pandemic in Western Germany

**DOI:** 10.3389/fpsyt.2021.648273

**Published:** 2021-04-23

**Authors:** Norbert Scherbaum, Udo Bonnet, Henning Hafermann, Fabrizio Schifano, Stefan Bender, Torsten Grigoleit, Jens Kuhn, Peter Nyhuis, Ulrich W. Preuss, Gerhard Reymann, Udo Schneider, Jo Shibata, Michael Specka

**Affiliations:** ^1^Department of Psychiatry and Psychotherapy, Medical Faculty, LVR-Hospital Essen, University of Duisburg-Essen, Essen, Germany; ^2^Klinik für Psychiatrie, Psychotherapie und Psychosomatische Medizin, Evangelisches Krankenhaus Castrop-Rauxel, Castrop-Rauxel, Germany; ^3^Psychopharmacology, Substance Misuse and Novel Psychoactive Substances Research Unit, University of Hertfordshire, Hatfield, United Kingdom; ^4^Klinik für Psychiatrie und Psychotherapie, LWL-Klinik Marsberg, Marsberg, Germany; ^5^Abteilung für Abhängigkeitserkrankungen, LVR-Klinik Langenfeld, Langenfeld, Germany; ^6^Klinik für Psychiatrie, Psychotherapie und Psychosomatik, Johanniter Krankenhaus Oberhausen, Oberhausen, Germany; ^7^Department of Psychiatry and Psychotherapy, University of Cologne, Cologne, Germany; ^8^Klinik für Psychiatrie, Psychotherapie und Psychosomatik, St. Marien Hospital Eickel, Herne, Germany; ^9^Vitos-Klinik für Psychiatrie und Psychotherapie, Herborn, Germany; ^10^Suchtmedizin, LWL-Klinik Dortmund, Dortmund, Germany; ^11^Medizinisches Zentrum für Seelische Gesundheit, Krankenhaus Lübbecke-Rahden, Lübbecke, Germany; ^12^Substitution Outpatient Clinic, Health Department of the City of Cologne, Cologne, Germany

**Keywords:** COVID-19, drug availability, cocaine, heroin, cannabis, novel synthetic opioids, novel psychotropic substances, pregabalin

## Abstract

**Background:** In response to the COVID-19-pandemic, a lockdown was established in the middle of March 2020 by the German Federal Government resulting in drastic reduction of private and professional traveling in and out of Germany with a reduction of social contacts in public areas.

**Research Questions:** We seek evidence on whether the lockdown has led to a reduced availability of illegal drugs and whether subjects with substance-related problems tried to cope with possible drug availability issues by increasingly obtaining drugs via the internet, replacing their preferred illegal drug with novel psychoactive substances, including new synthetic opioids (NSO), and/or by seeking drug treatment.

**Methods:** A questionnaire was anonymously filled in by subjects with substance-related disorders, typically attending low-threshold settings, drug consumption facilities, and inpatient detoxification wards from a range of locations in the Western part of Germany. Participants had to both identify their main drug of abuse and to answer questions regarding its availability, price, quality, and routes of acquisition.

**Results:** Data were obtained from 362 participants. The most frequent main substances of abuse were cannabis (*n* = 109), heroin (*n* = 103), and cocaine (*n* = 75). A minority of participants reported decreased availability (8.4%), increased price (14.4%), or decreased quality (28.3%) of their main drug. About 81% reported no change in their drug consumption due to the COVID-19 pandemic and the lockdown. A shift to the use of novel psychoactive substances including NSO were reported only by single subjects. Only 1–2% of the participants obtained their main drug via the web.

**Discussion:** Present findings may suggest that recent pandemic-related imposed restrictions may have not been able to substantially influence either acquisition or consumption of drugs within the context of polydrug users (including opiates) attending a range of addiction services in Germany.

## Introduction

In March 2020 the Federal Republic of Germany, in line with other states, put a lockdown strategy into effect as a response to the threat of the COVID-19 pandemic. The aim of this was to prevent new infections and to reduce stress on the health care system, especially the intensive care units (1). The lockdown included a drastic reduction of personal traffic by aircraft, car, or train across international borders, while the transport of commercial goods, e.g., by trucks and ships, within Germany and internationally was largely unaffected by these restrictions. From July 1, 2020, the restrictions regarding traveling were partially reduced both in Germany and in the European Union.

Given these restrictions within public and private life, one could argue whether the availability of illegal drugs was reduced in parallel with the COVID-19 pandemic. For example, cocaine and heroin available in Germany typically arrive from South America and Afghanistan, respectively. Within the context of a general reduction of international traveling, one could expect decreased trafficking of these drugs to Europe and to Germany in particular. As possible consequences of the reduced availability of certain drugs, higher prices, increased levels of contamination, and increased levels of risk/criminal behavior in order to obtain drugs were assumed (2). Moreover, it was expected that a higher number of drug addicts would claim access to therapeutic care and/or that they would increasingly utilize online sources of illicit drug delivery in order to compensate for decreased availability of illegal drugs on the street market (3). Within the context of online drug acquisition, a shift to novel psychotropic drugs (NPS) (4) as a substitute for common illicit drugs [e.g., synthetic cannabinoids as a substitute for cannabis, cathinones as a substitute for cocaine or amphetamines, and new synthetic opioids (NSO) such as fentanyl analogues as substitutes for heroin (5, 6)] could also be anticipated as a possibility.

Soon after the first lockdown measures had been introduced in most European countries, several studies were conducted on their impact on legal and illegal drug use. This included wastewater analyses in several large cities, which for example found decreased use of MDMA, amphetamines, and cocaine (7, 8). Other studies, for example, documented increased cannabis consumption by cannabis users (9), local shortages of heroin supply, or an increase in alcohol consumption (7). It is important to note that some results were heterogeneous and variable between places, drug types, and types of users investigated.

The principal aim of the present study was to collect data from users of illicit drugs, regarding the availability of their preferred substances within the context of the COVID-19 pandemic; in addition, we tried to ascertain participants' strategies for coping with the anticipated reduced drug availability; it was hypothesized here that these strategies included self-referral to addiction services, online purchase of drugs, and a shift to the use of remaining drugs, especially NPS and NSO. In order to investigate these issues, a survey was carried out on clients in contact with the drug addiction health care system, with a special focus on those clients currently using illegal drugs (e.g., those attending low-threshold services such as drug consumption facilities and detoxification units).

## Methods and Materials

For this multicenter investigation, 14 institutions were included, and 12 agreed to participate; most of these institutions had already collaborated in previous clinical addiction research projects (10). These 12 facilities included a drug consumption facility with an associated meeting point for clients (“crisis café”) (*n* = 1), a heroin prescription clinic (*n* = 1), inpatient detoxification wards (*n* = 10). In some of these institutions, the survey was also carried out in associated outpatient addiction services, e.g., opioid maintenance clinics (whose patients could be included if they concomitantly used illicit drugs). All facilities were situated in the Federal state North Rhine Westphalia: seven of them in the Ruhrgebiet, a metropolitan region; one in the large city of Cologne; and four (which recruited about one fifth of the sample analyzed) from smaller towns in rural areas.

For this survey a self-administered questionnaire with 37 items was designed. The questionnaire included questions regarding basic sociodemographic variables (age, sex), and presented a list of 15 legal or illegal psychotropic substances for which subjects should indicate the number of consumption days during the previous 30 days. The drugs presented were those identified as those used most frequently by drug users, in a comprehensive survey carried out recently (10). Subjects were then asked to identify their main drug (open question); regarding that main drug, they were then asked whether (a) its availability, price, or quality had changed after lockdown; (b) its use (with regard to frequency of use; shift to legal substances, including alcohol; shift to illegally acquired medications, such as benzodiazepines and pregabalin; shift to NPS and NSO) had changed; (c) a formal drug-related treatment (opioid maintenance or detoxification treatment) had been initiated, due to lockdown-related drug acquisition issues; and (d) drugs had been purchased online (ever purchased online, frequency of purchases, purchase for the first time during the lockdown). All these questions went with predefined answering options. To fill in the survey, subjects needed 10–15 min.

The survey was carried out between April 20 and September 9, 2020. The survey was developed by the addiction research team, partially based on the German version of the European Addiction Severity Index [EuropASI (11)] and discussed with single patients. A formal pilot phase was not carried out.

Participation was strictly anonymous and on a voluntary basis; no financial compensation for study participation was provided. In order to further guarantee respondents' anonymity regarding a survey presenting with drug acquisition/trafficking activities as a relevant topic, neither was a consent agreement signature requested, nor were participation rates or participants systematically recorded. The inclusion criterion was current (e.g., last 30 days) use of an illegal drug according to the German narcotics law; clients with no sufficient command of German or presenting with a manifest psychotic disorder were excluded. Ethical approval was granted by the ethics' committee of the University Hospital Essen (20-9350-BO).

Statistical analyses were carried out using descriptive statistics, in terms of absolute frequencies and percentages.

## Results

The total number of participants was 362. Out of these, 25 were excluded from data analysis, because the questionnaire was only partially filled in (*n* = 2), no main drug was indicated (*n* = 5), alcohol was indicated as the main psychoactive substance (*n* = 11), or a maintenance drug within maintenance treatment was identified as the main drug (*n* = 7).

The mean age of the 337 remaining clients was 38.5 (standard deviation [SD] 10.3); 262 (77.8%) were male. Most participants were multiple drug users (including illicit drugs, alcohol, and benzodiazepines, but excluding nicotine) with an average of 3.8 (SD 2.1; median 2) different substances used during the previous 30 days. Participants indicated as their main drug cannabis (*n* = 109), heroin (*n* = 103), cocaine (*n* = 75), amphetamines (*n* = 34), benzodiazepines (*n* = 8), pregabalin (*n* = 3), NPS (*n* = 3), Kratom (*n* = 1), or MDMA (*n* = 1). The largest proportion of participants was from in-patient drug detoxification facilities (*n* = 178; 52.8 %), followed by low-threshold facilities (drug consumption facility, associated counseling café, or heroin prescription clinic; *n* = 127, 37.7%), the remaining (*n* = 32, 9.5%) were from different settings, for example, maintenance clinics or out-patient services for the treatment of cannabis addiction.

Data from the three largest groups with respect to their main drug (heroin, cannabis, and cocaine) were further analyzed. The first set of statements concerned the availability of the main drug, its quality, and its price during the present COVID-19 pandemic (see Table 1). For all three main drugs, more than 80% of the subjects evaluated the availability of their main drug as unchanged compared with the situation before the lockdown. Conversely, only a small minority (heroin 10.8%, cannabis 8.3%, and cocaine 5.4%) reported that the availability of their main drug was reduced. About a third of the participants evaluated the quality of heroin (36.3%) and cocaine (34.2%) as having been reduced. Conversely, only 16.7% of the subjects reported a reduction in the quality of cannabis. About 75% in total (heroin 75.7%, cannabis 77.6%, and cocaine 74.3%) indicated that the price of their main drug was unchanged as compared to the pre-lockdown period, while <20% (for heroin and cannabis) and 10% (for cocaine) evaluated the price as having increased.

**Table 1:** Availability, price, and quality of the main drug.

			**Main drug**			
			**Heroin (*n* = 103)**	**Cannabis (*n* = 109)**	**Cocaine (*n* = 75)**	**Total (*n* = 287)**
Availability	Unchanged	*n*	84	90	61	235
		%	82.4%	82.6%	82.4%	82.5%
	Drug more accessible than before	*n*	1	0	2	3
		%	1.0%	0.0%	2.7%	1.1%
	Drug less accessible than before	*n*	11	9	4	24
		%	10.8%	8.3%	5.4%	8.4%
	Fluctuating levels of access	*n*	6	10	7	23
		%	5.9%	9.2%	9.5%	8.1%
Price	Unchanged	*n*	78	83	55	216
		%	75.7%	77.6%	74.3%	76.1%
	Decreased	*n*	3	5	4	12
		%	2.9%	4.7%	5.4%	4.2%
	Increased	*n*	20	15	6	41
		%	19.4%	14.0%	8.1%	14.4%
	Fluctuating	*n*	2	4	9	15
		%	1.9%	3.7%	12.2%	5.3%
Quality	Unchanged	*n*	65	90	48	203
		%	63.7%	83.3%	65.8%	71.7%
	Worse	*n*	37	18	25	80
		%	36.3%	16.7%	34.2%	28.3%

*Note that some responses were missing for some questions*.

The second set of statements related with changes in the pattern of drug use associated with the COVID-19 pandemic (see Table 2). Most subjects (e.g., heroin users, 83%; cannabis users, 84.1%; and cocaine users, 73%) evaluated the frequency of the use of their main drug as unchanged compared with the period before the lockdown. Some 27% of clients for whom cocaine was the main drug reported a reduced frequency of drug use. Only some 10% of clients reported a shift to an increased use of legal substances, mainly alcohol. About 5% (*n* = 14) reported a shift toward illegally acquired medications such as benzodiazepines, pregabalin, or opioid maintenance drugs. Only a very small number of participants reported a shift to the use of NPS: one client shifted from cannabis as the main drug to synthetic cannabinoids and another shifted from the main drug heroin to NSOs. None of the participants reporting cocaine as their main drug shifted to the use of novel synthetic stimulants, such as cathinones, or “bath salts,” etc.

**Table 2:** Shift to other substances and initiation of a formal drug treatment because of problems with availability of the main drug during the COVID-19 pandemic.

		**Main drug**			
		**Heroin (*n* = 103)**	**Cannabis (*n* = 109)**	**Cocaine (*n* = 75)**	**Total (*n* = 287)**
No change in consumption of main drug	*n*	83	90	54	227
	%	83.0%	84.1%	73.0%	80.8%
Shift to legal substances (alcohol)	*n*	12	8	6	26
	%	12.0%	7.5%	8.1%	9.30%
Shift to illegally acquired medications, maintenance drugs	*n*	8	2	3	13
	%	8.0%	1.0%	4.1%	4.6%
Shift to synthetic cannabinoids	*n*	0	1	1	2
	%	0.0%	0.9%	1.4%	0.7%
Shift to synthetic stimulants	*n*	0	2	0	2
	%	0.0%	1.9%	0.0%	0.7%
Shift to new synthetic opioids	*n*	1	0	0	1
	%	1.0%	0.0%	0.0%	0.4%
Started a new episode of opioid maintenance treatment because of COVID-19 pandemic	*n* %	7 7.0%	4 3.7%	0 0.0%	11 3.9%
Started detoxification treatment because of COVID-19 pandemic	*n*	6	5	5	16
	%	6.0%	4.7%	6.8%	5.7%

*Note that some responses were missing for some questions*.

A third set of statements concerned the initiation of treatment in the context of the COVID-19 pandemic as a consequence of changed availability or price in the context of the lockdown. In total, 8% of participants reported that they had started detoxification and/or had initiated maintenance treatment due to the COVID-19 situation.

The fourth set of items concerned the possible shift from street trafficking of drugs to an increase of drug ordering via smartphone or personal computer (see Table 3). Some 50–65% of participants reported having internet access, but only a minority of <10% reported having ever purchased drugs online. About five participants had acquired illicit drugs online more than five times, and one of them reported having carried out such online purchase activities more than 100 times in his/her lifetime. Conversely, online acquisition of illicit drugs during the pandemic was only carried out by single individuals, i.e., one subject reporting cannabis as the main drug and one subject reporting cocaine.

**Table 3:** Online acquisition of illicit drugs.

			**Main drug**			
			**Heroin (*n* = 103)**	**Cannabis (*n* = 109)**	**Cocaine (*n* = 75)**	**Total (*n* = 287)**
Internet connection available to the subject		*n*	64	72	39	175
		%	64.0%	67.3%	53.4%	62.5%
Ever purchased drugs over the internet		*n*	10	6	5	21
		%	10.0%	5.6%	6.8%	7.5%
Purchase of main drug over the internet during the pandemic	No	*n* %	98 99.0%	105 97.2%	72 98.6%	275 98.2%
	Yes, for the first time	*n*	0	1	1	2
		%	0.0%	0.9%	1.4%	0.7%
	Yes, same frequency as before	*n*	1	1	0	2
		%	1.0%	0.9%	0.0%	0.7%
	Yes, more frequently	*n*	0	1	0	1
		%	0.0%	0.9%	0.0%	0.4%

*Note that some responses were missing for some questions*.

## Discussion

The federal state of North Rhine Westphalia, in which the present study was conducted, is a densly populated region in the Western part of Germany. About 10% of its 11 million residents aged 18–64 years show risky alcohol consumption (>12 g alcohol daily in women, >24 g in men) (12). The total 12 month prevalence of illegal drugs is 7.9%, most frequently cannabis (6.5%) but also amphetamines and methamphetamine (1.1%), MDMA/ecstasy (0.8%), novel psychoactive substances (NPS, including synthetic opioids), heroin (0.4%), or cocaine (1.0%). It was estimated that 1.2% of the population aged 18–64 years show a dependency [according to DSM-IV (13)] on one or more illicit drugs during a year, including 1.1% for cannabis, and 0.4% show substance misuse. Besides cultivation within the state, cannabis is supplied mainly through importation from the neighboring Netherlands, where a considerable share of the consumed amphetamine, methamphetamine, and MDMA is also produced; important routes for the supply of heroin and cocaine from outside of Europe are via the large ports and airports of Belgium and the Netherlands (14). It was anticipated that lockdown measures and closing of borders would influence the quantity and quality of illicit drugs for users in contact with the drug treatment and low-threshhold services for drug addicts.

### Summary of the Findings

In the present study, about 80% of the subjects did not report a reduced availability or an increased price of the illegal drugs heroin, cocaine, or cannabis. The quality of these drugs was evaluated as worse by 28.3% as compared with the period before the lockdown. Furthermore, only a small minority switched from their main drug to legal drugs, especially alcohol, or to illegally acquired medications such as benzodiazepines or gabapentinoids. Only one subject whose main drug was cannabis switched to synthetic cannabinoids, one heroin addict switched to NSOs, and only a few subjects initiated treatment due to a reduced availability of their main drug. In our sample, the lifetime experience of ordering illegal drugs online was low, e.g., <10%, and only two subjects ordered their main drugs for the first time during the COVID-pandemic.

Basic sociodemographic and clinical data (e.g., age around 40; mostly males; and typically polydrug users) of the present sample are consistent with the description of samples of illegal drug-using clients attending German drug services (10); in two recently published investigations carried out in Western parts of Germany addiction clients confirmed that heroin, cocaine, and cannabis were their main illegal drugs (10, 15), with very limited numbers of clients reporting NPS and NSO intake. Typical low-threshold addiction facilities' clients polydrug, including opiates, users. However, drug addicts might define cocaine or cannabis as their main (illegal) drug, especially in the case of maintenance treatment. However, consistent with recent inpatient detoxification treatment data from Western Germany (10), a growing problem of gabapentinoid misuse, predominantly among opioid addicts (16–18), with three participants having identified pregabalin as their main drug, was highlighted here.

### Comparison With Previous Covid-19-Related Findings

Current results are not fully consistent with recent findings about drug abuse during the COVID-19 pandemic (7, 19). In April and May 2020, EMCDDA carried out the “European Web Survey on Drugs: COVID-19” (EWSD-COVID), in which more than 10,000 subjects of at least 18 years of age were asked about their use of illicit drugs (7). Some 46% of respondents reported a reduced use or no drug use during the lockdown. In particular, 20% of cocaine or MDMA users reported to have stopped the use of one of these drugs during the pandemic. In contrast, among current users of illegal drugs (defined as drug consumption during the last 12 months) some 25% reported an increased drug use, especially of cannabis (about 15%) and of alcohol (about 15%). Conversely, the EMCDDA expert opinions regarding availability and price of drugs, albeit not supported by empirical evidence, yielded a heterogeneous picture, with different situations in the different EU countries. Indeed, the price of cannabis was suggested to have increased in several EU member states, in parallel with a decrease in its availability. However, the European Web Survey focused on users of illicit drugs, not only on subjects with a clear drug addiction status.

A further document, elaborated by the EMCDDA in cooperation with Europol (19), concluded that the European drug supply scenario had not significantly reduced during the Covid-19 pandemic. In fact, while air trafficking was vastly reduced, the transport of commercial goods by ship, air freight, and so on had somehow continued during the pandemic, and this may have facilitated the transportation of drugs such as cocaine and heroin. In addition, the domestic production of cannabis in some European countries was not restricted by the COVID-19 pandemic (19). These issues may explain the lack of an overall significant reduction of drug supply during the pandemic, although there may have been illegal drug acquisition issues in some places. In Germany, this overall scenario was confirmed by the Federal Criminal Police Office (Ms. B. Hübner, spokeswoman for the Federal Criminal Police Office). In addition, even during the lockdown, several important drug seizures were successfully carried out in EU countries (18).

Lockdown measures made it more difficult to meet with dealers and friends and this may have led to a breakdown of the local street market for drugs. This could have facilitated the occurrence of other forms of drug trafficking, especially buying of illegal drugs online and delivery of drugs by post and parcel services. According to the EMCDDA (19), however, there was only a small increase in drug buying from the darknet during the pandemic. In our sample of polydrug addicts, including users of opiates, only 2/3 of respondents had internet access at all. Consistently with this, online drug acquisition activities during the pandemic were carried out here only by single individuals. Difficulties handling the web and especially the darknet (20, 21) with the related money transfer issues may have limited the availability of the online acquisition option in the current population of marginalized polydrug drug addicts with minimal resources. Conversely, the online option, which may well-include access to messenger services facilitating drug orders and deliveries, may be an easier option for those with a regular income and a routine use of the web. It must be stressed, though, that during lockdown, the internet and the world wide web also offered opportunities for continued “telehealth” care for patients with mental health issues, including those with substance use problems. This extends to online individual or group therapy (22, 23).

A significant reduction of the clients' main drug availability level was not here reported and this may have arguably reduced the need for a shift to NPS/NSO use. Consistent with this, recent data (10, 15) suggested that while about 40% of drug addicts open to addiction services had a lifetime experience with NPSs—and especially with synthetic cannabinoids—this use was sporadic, due to the often severe side effects experienced, which are a strong argument against repeated levels of use even for experienced drug addicts. As for NSOs, only one heroin user shifted here to the use of these substances; this is fully consistent with recent German data (15), but it contrasts with reports from the USA, where an opioid epidemic is occurring (5). There was also only a small shift toward more alcohol use. Previous studies in the general population in the United States or elsewhere found no sustained increase of alcohol use (24) or even decreases due to the discontinuation of social drinking events (8), and on the individual level, large proportions of subjects either increased or decreased their alcohol use during the pandemic. It must be stressed that in the present study increased alcohol use *per se* was not investigated, but rather the COVID-19-related shifts away from the main drug.

Finally, although long-term follow-up German studies have suggested that on and off treatment episodes alternate in the life of opiate addicts (25), the substantially unchanged levels of drug availability did prompt the need for the initiation of a new treatment (e.g., maintenance or detoxification treatment) episode in only <10% of interviewees.

### Limitations

Only a minority of subjects from the participating inpatient detoxification wards and some 50% of those attending drug consumption/low-threshold facilities participated in the survey, and this may limit the generalizability of current finding. According to the study design, questionnaires were handed out to those subjects who satisfied the study inclusion criteria; however, to respect anonymity, there were no specific checks to assess whether questionnaires were *de facto* filled in by the individuals themselves. No measures were taken here to increase the response rate. In addition, the main drug was self-reported by the interviewees, not by the clinician. However, current sociodemographic and clinical data were here fully consistent with those characterizing samples taken from addiction services in Germany (10).

## Conclusions

Current findings may support the idea that at least in the first part of 2020 the pandemic-related imposed restrictions may not have been able to substantially influence the demand, acquisition, and consumption of drugs within a context of polydrug users, including users of opiates, attending a range of addiction services in Germany. Further studies, focusing on the issues relating to the persistence of the current pandemic, should be carried out to assess the impact of confinement on these vulnerable clients drug intake.

## Data Availability Statement

The raw data supporting the conclusions of this article will be made available by the authors, without undue reservation.

## Ethics Statement

The studies involving human participants were reviewed and approved by Ethikkommission des Universitätsklinikums Essen, Robert-Koch-Str. 9-11, 45147 Essen, Germany ethikkommission@uk-essen.de. Written informed consent for participation was not required for this study in accordance with the national legislation and the institutional requirements.

## Author Contributions

NS, MS, and HH planned and designed the empirical study. NS, UB, HH, SB, TG, JK, PN, UP, GR, and JS planned the data collection and participated in defining and accessing the study population. NS, UB, FS, and MS wrote the manuscript. SB, TG, JK, PN, UP, GR, and JS reviewed the manuscript. All authors contributed to the article and approved the submitted version.

## Conflict of Interest

NS received honoraria for several activities (advisory boards, lectures, manuscripts) from the factories AbbVie, Camurus, Hexal, Janssen-Cilag, MSD, Medice, Mundipharma, Reckitt-Benckiser/Indivior, and Sanofi-Aventis. During the last three years he participated in clinical trials financed by the pharmaceutical industry. The remaining authors declare that the research was conducted in the absence of any commercial or financial relationships that could be construed as a potential conflict of interest. The reviewer SC declared a shared affiliation with one of the authors, FS to the handling editor at time of review.
