# Supplementary material for: Availability of Illegal Drugs During the COVID-19 Pandemic in Western Germany
Source: Front Psychiatry. 2021 Apr 23;12:648273. doi: 10.3389/fpsyt.2021.648273 (PMC8102785; doi:10.3389/fpsyt.2021.648273)
Supplement: Supplementary file 1 [file Data_Sheet_1.PDF]

Illegale Suchtmittel und die Corona-Virus-Epidemie

Datum: _______________ Ort: ________________________

Alter: ☐☐ Jahre Geschlecht: ☐ männlich ☐ weiblich

Umstände der Befragung

☐ Entzugsstation ☐ Substitutionsambulanz ☐ Suchtambulanz

☐ Drogenkonsumraum ☐ Krisencafé ☐ andere Umstände

Konsummuster

An wie vielen der letzten 30 Tage haben Sie folgende Suchtmittel konsumiert?

Alkohol: ☐☐

Benzodiazepine: ☐☐

Cannabis: ☐☐

Synthetisches Cannabis („Spice" und ähnliche): ☐☐

Amphetamine: ☐☐

Ecstasy: ☐☐

Neue synthetische Stimulanzien

(„Badesalz", „Research Chemicals" o.ä): ☐☐

Heroin: ☐☐

Buprenorphin / Methadon / Polamidon etc.

…als ärztlich verschriebene Substitutionsmittel: ☐☐

Opiathaltige Schmerzmittel (z. B. Tramal, Tilidin etc.): ☐☐

Neue synthetische Opioide (z. B. Fentanyl und Verwandte): ☐☐

Pregabalin, z.B. Lyrica (nicht ärztlich verschrieben) ☐☐

Kokain ☐☐

Andere Drogen, wenn ja, welche? ___________________ ☐☐

<u>Verfügbarkeit der Hauptdroge</u>

Sie konsumieren in abhängiger Weise mindestens ein Suchtmittel. Vor dem Hintergrund der Corona-Epidemie möchten wir Sie fragen, ob sich die Verfügbarkeit Ihrer Hauptdroge seit Mitte März 2020 (Verkündigung der Einschränkungen im öffentlichen Leben) verändert hat.

Bitte geben Sie hierfür zunächst den Namen Ihrer Hauptdroge an (Sofern Sie in Substitutionsbehandlung sind, geben Sie bitte an, welche illegale Droge für Sie in den letzten Monaten, also auch vor Ausbruch der Corona-Virus-Epidemie, am wichtigsten ist).

____

Inwieweit hat sich die Verfügbarkeit Ihrer Hauptdroge seit Mitte März 2020 verändert (bitte Zutreffendes ankreuzen)?

- ☐ Meine Hauptdroge ist genauso verfügbar wie zuvor.
- ☐ Meine Hauptdroge ist leichter verfügbar (ich benötige weniger Zeit als zuvor zur Beschaffung meiner Hauptdroge).
- ☐ Meine Hauptdroge ist weniger verfügbar als zuvor (ich benötige mehr Zeit als zuvor zur Beschaffung meiner Hauptdroge).
- ☐ Meine Hauptdroge war in den letzten Wochen wechselnd verfügbar.

Qualität der Hauptdroge

- ☐ Die Qualität meiner Hauptdroge ist unverändert.
- ☐ Die Qualität meiner Hauptdroge ist in den letzten Wochen deutlich schlechter als vorher (zum Beispiel: weniger wirksam, mehr Verschnitt, unangenehme Beimischungen)

Preis der Hauptdroge

Inwieweit hat sich der Preis Ihrer Hauptdroge seit der Verkündigung der Einschränkungen des öffentlichen Lebens Mitte März 2020 verändert?

- ☐ überhaupt nicht
- ☐ Der Preis ist geringer geworden
- ☐ Der Preis ist gestiegen
- ☐ Der Preis unterlag in den vergangenen Wochen erheblichen Schwankungen.

Veränderung des Konsummusters
Unter einer Veränderung von Verfügbarkeit und Preis unter den Corona-bedingten Einschränkungen könnte sich Ihr Konsum von Suchtmitteln verändert haben.

Hat sich Ihr Konsum Ihrer Hauptdroge bzw. anderer Suchtmittel seit März verändert? (Zutreffendes bitte ankreuzen; gegebenenfalls auch mehrere Antworten als für Sie zutreffend ankreuzen)

- ☐ nein (ich nehme meine Hauptdroge etwa so häufig ein wie vor den Corona-Einschränkungen)
- ☐ Ich nehme meine Hauptdroge in einer geringeren Menge oder an weniger Tagen ein.
- ☐ Es gibt eine Verlagerung des Suchtmittelkonsums zu legal verfügbaren Suchtmitteln, insbesondere Alkohol, die ich jetzt häufiger einnehme.
- ☐ Es gibt eine Verlagerung zu nicht ärztlich verschriebenen Medikamenten, z.B. Substitutionsmittel (Methadon, Polamidon, Methaddict usw.), Benzodiazepine, Pregabalin (z.B. Lyrica) oder andere

  Falls zutreffend, bitte entsprechende Substanz benennen: ____________________________

- ☐ Ich habe nach März 2020 mit einer Substitutionsbehandlung begonnen, da sich die Verfügbarkeit und/oder der Preis meiner Hauptdroge verändert hat.
- ☐ Ich habe wegen der Corona-bedingten Veränderungen von Verfügbarkeit und/oder Preis meiner Hauptdroge eine Entzugsbehandlung aufgenommen.
- ☐ Es fand eine Verlagerung der Einnahme in Richtung auf neue Drogen („Legal Highs", „Research Chemicals") statt.

  Falls zutreffend, bitten entsprechende Substanz ankreuzen)

  - ☐ Synthetische Cannabispräparate („Spice" o.ä.)
  - ☐ Neue synthetische Stimmulanzien („Badesalz", „Reserch Chemicals" o.ä.)
  - ☐ Neue synthetische Opioide (Fentanyl und Verwandte).

Veränderung der Drogenbeschaffung

Haben Sie jemals Ihre Hauptdroge über das Internet bestellt?
☐ ja ☐ nein
Falls ja, etwa wie häufig? ____mal

Verfügen Sie aktuell (zumindest in den letzten 3 Monaten) über einen PC/Smartphone, mit dessen Hilfe Sie theoretisch über das Internet Ihre Hauptdroge bestellen könnten?

☐ ja ☐ nein

Haben Sie unter den Einschränkungen der Corona-Virus-Epidemie Ihre Hauptdroge über das Internet bestellt? (bitte Zutreffendes ankreuzen)
☐ nein
☐ ja, zum ersten Mal
☐ ja, so wie auch schon früher
☐ ja, aber häufiger als vor der Corona-Virus-Epidemie

Haben Sie noch Kommentare und Ergänzungen?
(Insbesondere wenn nicht Ihre Hauptdroge, aber eine andere von Ihnen regelhaft konsumierte Droge sich in Preis, Verfügbarkeit etc. während der Corona-Virus-Epidemie verändert hat.)

Wir danken Ihnen für Ihre Teilnahme an unserer Befragung!
